# Editorial: Innovative pharmacometric approaches to inform drug development and clinical use

**DOI:** 10.3389/fphar.2023.1274139

**Published:** 2023-10-04

**Authors:** Maria Garcia-Cremades, Zinnia P. Parra-Guillen, Victor Mangas-Sanjuan, Hugo Geerts

**Affiliations:** ^1^ Department of Pharmaceutics and Food Technology, School of Pharmacy, Complutense University of Madrid, Madrid, Spain; ^2^ Institute of Industrial Pharmacy, Complutense University of Madrid, Madrid, Spain; ^3^ Pharmaceutical Science Department, School of Pharmacy and Nutrition, University of Navarra, Pamplona, Spain; ^4^ IdiSNA, Navarra Institute for Health Research, Navarra, Spain; ^5^ Department of Pharmacy and Pharmaceutical Technology and Parasitology, Faculty of Pharmacy, University of Valencia, Valencia, Spain; ^6^ Interuniversity Research Institute for Molecular Recognition and Technological Development, Valencia, Spain; ^7^ Certara, Princeton, NJ, United States

**Keywords:** pharmacometrics, quantitative systems pharmacology, clinical trials, machine learning, POPPK

A successful Drug Development is driven by many factors, but ultimately it is often dependent upon the weakest part. Therefore, it is necessary to utilize all tools available to support the complete journey for a successful development program. Model-informed drug development (MIDD) in which crucial information is generated from mathematical analysis or modeling, is rapidly becoming a powerful tool in pharmaceutical R&D and in the regulatory environment. Appropriate use of modeling can contribute to more rational and efficient decision-making in drug development, leading to substantial resource savings and shortened timelines.

MIDD can broadly be divided in data- and mechanism-driven modeling and the manuscripts in this Research Topic are good examples of the diversity of applied algorithms. In addition, the large range of disease indications is testimony to the impact of these modeling techniques in clinical drug development and clinical practice.

Data-driven approaches include more traditional statistical bio-informatics analyses of large datasets or machine learning algorithms to derive predictive insights (Ribba et al.). The quality of these predictions is heavily dependent upon the nature of the training sets and issues of generalizability need to be addressed.

Two articles look to derive estimates of drug exposure using more advanced Physiologically-based Pharmacokinetic models (PBPK) to predict drug exposure in other populations (Zazo et al.). An interesting combination of traditional PopPK modeling with machine learning aims to understand the role of covariates (Zhu et al.). Finally, PK modeling can also be used to derive the quantitative pharmacokinetics trajectory of biomarkers which can clarify the contribution of these biomarkers in clinical development (Michelet et al.).

Mechanism-driven modeling on the other hand can be helpful in those cases where data are lacking or noisy. This is often the case in CNS disorders, where robust quantitative biomarkers are scarce, functional scales are often based on structured interviews and biomarkers are often not strongly related to clinical outcomes. This section includes a contribution, using computational neuroscience to gain insights in the mechanisms of catatonia (Roberts and Conour) and a position paper on computational psychiatry, especially with regard to reward physiology (Ribba), a field in full development. This underscores the power of combining the academic discipline of computational neurosciences with Quantitative Systems Pharmacology.

Another paper describes the powerful prediction capability for combination therapy and virtual patient modeling in oncology (Anbari et al.). Here the authors use a mechanistic modeling of different therapeutic modalities, each calibrated with their own clinical dataset, to explore the optimal conditions for combination therapy and to estimate the variability that can provide estimates for power calculations.

In summary, Model-Informed Drug Development (MIDD) is rapidly becoming an essential tool for quantitatively assessing the relevance of data- and knowledge-based information to support not only clinical trial development but also clinical practice. From estimation of effective doses over combination therapy to personalized medicines, this approach has matured to the point that they can make the difference between a successful and a failed clinical development project.

